# Raising of Anillin expression in para-cancerous hepatocytes is associated with hepatic depolyploidization and short-term recurrence of hepatocellular carcinoma after radical operation

**DOI:** 10.7150/jca.72890

**Published:** 2022-05-29

**Authors:** Nan Wang, Fengjie Hao, Yifan Zhang, Wen Xu, Yongjun Chen, Xiaochun Fei, Junqing Wang

**Affiliations:** 1 Department of General Surgery, Ruijin Hospital, Shanghai Jiao Tong University School of Medicine, 197, Rui Jin Er Road, Shanghai 200025, People's Republic of China; 2 State Key Laboratory of Bioreactor Engineering and Shanghai Key Laboratory of New Drug Design, School of Pharmacy, East China University of Science and Technology, Shanghai, 200237, People's Republic of China; 3 Department of Pathology, Ruijin Hospital, Shanghai Jiao Tong University School of Medicine, 197, Rui Jin Er Road, Shanghai 200025, People's Republic of China

**Keywords:** Anillin, depolyploidization, karyoplasmic ratio, tumor recurrence, HCC

## Abstract

**Background:** Short-term recurrence of hepatocellular carcinoma (HCC) after radical operation results in poor prognosis and short overall survival period. Assessment of the post-operational recurrence risk could provide an advantage for preventing lethal progress by assisting to set up necessarily individual adjuvant treatment. Here, on basis of our previous research on the ploidy status of hepatocytes, we detected the expression of Anillin, a pivotal regulating gene of depolyploidization in para-cancerous hepatocytes, and discussed the relation between its alternative expression and short-term recurrence of HCC patients after radical operation.

**Methods:** One hundred and twenty-one specimens of the para-cancerous tissues from a cohort of HCC patients were collected. The RT-qPCR assay and the Immunohistochemistry assay were conducted for a thorough profile of Anillin expression, along with the analysis of patients' information from the GEO database. The clinicopathological para-maters of the patients were detected to determine the relationship between Anillin in the para--cancerous and the tumor relapse. Specimens treated with HE staining were examined for the precise count of the binuclear polyploid hepatocytes, and the karyoplasmic ratio was calculated.

**Results:** Anillin was verified raised in the para-cancerous tissues of the patients who relapsed. The raising of Anillin in para-cancerous tissue is related to poor clinicopathologic features of HCC patients and short-term recurrence. Binuclear polyploid hepatocytes were reduced along with a higher expression of Anillin, and the proportion of high karyoplasmic ratio hepatocytes was significantly decreased.

**Conclusion:** The raising of Anillin is associated with the depolyploidization of hepatocytes in the representation of losing binuclear hepatocytes and reducing proportion reduction of high karyoplasmic ratio hepatocytes. Measuring Anillin in para-cancerous tissue, along with the pathomorphological examination may provide us a strategy for screening high risk of HCC recurrence, and help to design individual adjuvant treatment post-operation.

## Introduction

Hepatocellular carcinoma (HCC) counts for over 90% of human liver malignancies, ranking third in mortality worldwide as a great challenge for clinical doctors and researchers 1, 2. Radical surgery is the most important treatment for HCC. Whereas, surgical treatment is limited not only because of the later stages of HCC patients when diagnosed, but also the high rate of recurrence, metastasis, and chemoresistance. Even though in recent years, the advancement of the transformation therapy, including targeted therapy and immunotherapy, which provides the patients a precious opportunity for descending tumor stages and undergoing radical resection, short-term relapse *in situ* still occurred in about 20% of the patients, and strongly restricts the over-all survive 3, 4. On basis of this background, the clinical problem of evaluating and preventing tumor progress and recurrence presents to be a much more urgent issue for improving the systematic treatment.

As acknowledged, the basic components of human somatic chromosomes are diploid (2n), whereas a small portion of human organs and tissues are structured with cells containing more than two groups of homologous chromosomes, which are called polyploid cells (4n, 8n) 5. Commonly, polyploidy widely exists in plants and fish. In note, polyploidy is also found in mammalian organs, such as skeletal muscle, brain, heart, and liver, and the complex functions of these cells with balanced duplications of all chromosomes in different microenvironments remain largely unknown 6.

In the liver, polyploid is a defining characteristic, which is observed in up to 90% of mice and 40% of human hepatocytes 7, 8. Accumulating evidence has been reported that polyploid exerts pivotal bio-functions in the liver, including undertaking complex and tremendous physiological processes, promoting tissue regeneration after chronic liver injury, and buffering chromosome aberration referring to tumorigenesis 9, 10. Recent research has gradually revealed the importance of the polyploidy status of hepatocytes in effectively repairing normal liver tissue such as liver resection, and in preventing tumorigenesis induced by acute or chronic liver injury and inflammation 11, 12.

On basis of our previous research, the actin-binding protein Anillin has been verified anomalously overexpressed in HCC tumor tissues and cells and induces tumor cell division and growth. Anillin is also a pivotal regulating gene triggering cell mitosis through modulating cytokinesis, which is the final step of cell division and determines the ploidy status of hepatocytes physiologically 13, 14. In brief, as described by Zhu, et.al, the binuclear polyploid hepatocytes compose the main population of the polyploid hepatocytes under normal physiological conditions, and depletion of Anillin in hepatocytes could theoretically reduce binuclear hepatocytes and facilitate tumorigenesis consequentially 15. These findings strongly illustrated us, and we wonder to detect the relation between Anillin and the proportion of binuclear hepatocytes during the process of HCC, and also the correlation with the post-operation recurrence, which is a point that has not been discussed before. Notably, the ploidy status of the tumor tissues is aberrantly and mingled with numerous aneuploidy tumor cells, and is excluded for evaluation.

In this study, we collected paired HCC specimens and detected the expression profile of Anillin, especially in the para-cancerous liver tissues. We evaluated the correlation between para-cancerous Anillin expression and the incidence of tumor recurrence. Meanwhile, the binuclear polyploid hepatocytes and the relative karyoplasmic ratio were calculated for further discussion of the potential correlation either with Anillin or tumor recurrence. The results of our study indicate that the proportion of binuclear polyploidy hepatocytes and the portion of large karyoplasmic hepatocytes are negatively correlated with the expression of Anillin in the para-cancerous liver tissue, which prompts a possibility for evaluating the high risk of recurrence after radical resection.

## Materials and methods

### Clinical specimens

Paired specimens including the tumor tissues and the para-cancerous liver tissues were collected from a 121 HCC patients cohort, which was performed radical resection without accepting any preoperative and post-operational therapies, at the Department of General Surgery, Ruijin Hospital, Shanghai Jiao Tong University School of Medicine (2018 to 2019). All the specimens should include tumor tissue and a broad non-cancerous margin more than 1cm to the bord of the tumor, for the comparison of the para-cancerous tissues close to the tumor and the distant ones. Specimens were arranged for hematoxylin-eosin (HE) staining regular for the basic pathological exam. Informed consent was obtained, and the study was approved by the Ethics Committee of Ruijin Hospital, Shanghai Jiaotong University School of Medicine. Simultaneously, tissues were made into tissue microarray by Outdo Biotech Company, Shanghai, China. The clinicopathological features were collected, including gender, age, tumor size, the number of lesions, grades et.al. And the patients were divided into two groups according to the short-term recurrence within 2 years post-operation: the Recurrence group and the No-recurrence one.

### RT-qPCR assay

RNA isolation was conducted from the specimens respectively according to the instruction of the TRIzol reagent (Invitrogen, USA). The first-strand cDNA was synthesized by using the High-Capacity cDNA Reverse Transcription Kit (ABI, USA), and the primers of the mRNAs were synthesized by Shanghai JIKE Biotech Company (Shanghai, China) (Supplementary Table 1). Real-time quantitative polymerase chain reaction (RT-qPCR) was conducted according to the TaqMan Gene Expression Assays protocol (ABI, USA).

### Immunohistochemistry assay

The antibody against Anillin was purchased from Abcam, USA. The Immunohistochemistry assay was carried out following our previously described methods 16. In brief, the para-cancerous tissue sections with 4μm thickness were cut from paraffin-embedded tissue blocks, deparaffinized and rehydrated, and treated with 0.01mol/l citrate buffer (pH 6.0) for antigen retrieval. After blocking with goat serum solution for 45 min, the sections were incubated with primary antibodies at 4℃over-night. Antibodies used for IHC included antibodies against Anillin (1:200, Abcam, USA). After washing thrice with PBS, sections were incubated with biotin-labeled secondary immunoglobulin (1:100, DAKO, Glostrup, Denmark) for 1h at room temperature. The sections were then stained with diaminobenzidine (DAB, DAKO, Glostrup, Denmark) and were re-stained with hematoxylin at room temperature. Two experienced pathologists were assigned independently and blindly for detecting Anillin expression through IHC assay. The specimens were separated into two groups according to the staining intensity grade: no to low staining (0∼1+) and moderate to high staining (2+∼3+).

### HE staining examination and karyoplasmic ratio calculation

Specimens of the para-cancerous tissues were set up for HE staining examination along with the tumor tissues in regular and were assigned to two experienced pathologists independently for detection. The binuclear hepatocytes were counted and excluded with the interstitial cells and connective tissue. The proportion of the binuclear hepatocytes was calculated. Meanwhile, the karyoplasmic ratio of the individual cell ranging 1 cm close to the tumor margins was calculated. The cutoff value of the karyoplasmic ratio was set into two groups: ≥0.8, and <0.8, and the proportion of the large karyoplasmic ratio hepatocytes was calculated.

### Analysis of the HCC dataset from the GEO database

Gene Expression Omnibus (GEO, https://www.ncbi.nlm.nih.gov/geo/) datasets including HCC patients' information were reviewed, and GSE45114 was screened out according to its comprehensive recurrence information. The gene expression profile of the GSE45114 dataset was downloaded, and the platform of it is GPL5918 (CapitalBio Human 22k oligonucleotide microarray).

### Statistical analysis

Statistical analysis was conducted by using SPSS 20.0. *P*-values were calculated following an unpaired Student's *t*-test and Fisher's exact test. Spearman correlation analysis and Logistic regression analysis were used to describe the correlation between the various parameters and the risk of short-term recurrence. Differences were considered statistically significant at* P*-values < 0.05.

## Results

### Rise in Anillin was detected in the para-cancerous tissues of the patients recurred in short-term after radical resection, and correlated with dismal clinicopathologic features

Our previous research demonstrated that Anillin was anomalously over-expressed in both HCC tumor tissues and cell lines. Interestingly, we also observed that a small portion of the para-cancerous liver tissue presented a relatively highly Anillin expression. However, in our previous study, any further clinicopathologic significance of this gene in the non-cancerous tissues has not been assessed yet. Meanwhile, a small number of the cases limited the further analysis.

**Fig 1:**
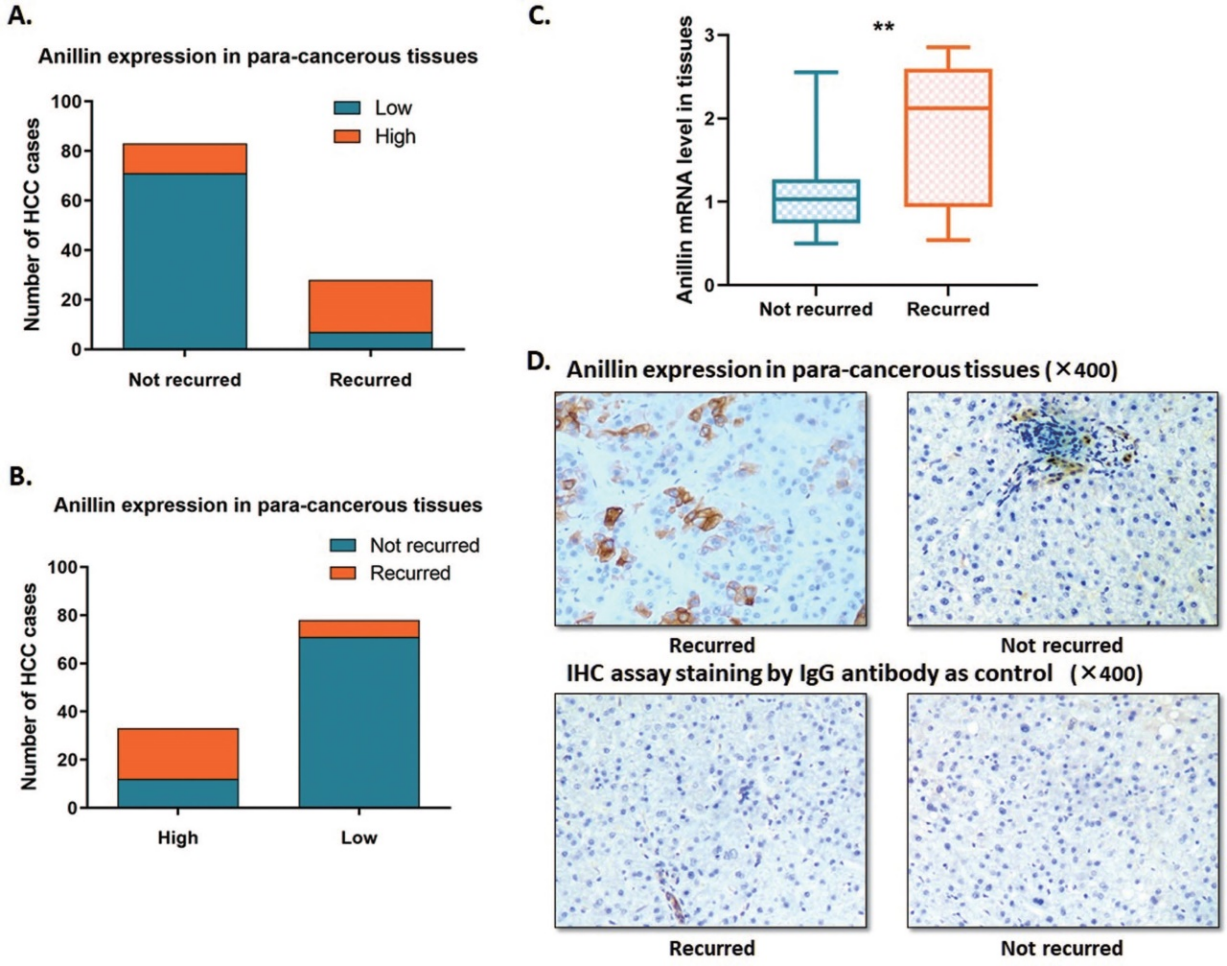
** Anillin expression profile in the para-cancerous liver tissues.** Statistic of the number of cases with relatively higher or lower Anillin expression in the para-cancerous liver tissues. **A~B.** 28 out of 121 cases recurred within 2 years after the operation. 33 cases presented a relatively higher Anillin expression in the para-cancerous liver tissues. Among them, 63.6% (21/33) recurred in the short term. Only 7.95% (7/88) cases presented lower Anillin in para-cancerous tissues without short-term recurrence (*P*<0.01). **C.** RT-qPCR assay demonstrated a significantly higher expression of Anillin mRNA in the para-cancerous tissues with short-term recurrence (**P<0.01). **D.** Representative graph of immunohistochemistry analysis (400✕) of the 121 HCC cases. Specimens stained IgG anti-body was regarded as control. Para-cancerous tissue Anillin expression in the recurred cases was significantly higher than in those without recurrence.

In this study, we expanded the sample size of HCC cases. The RT-qPCR and the IHC assay were conducted to indicate the Anillin expression profile in the para-cancerous liver tissues. Around 23.1% (28/121) of cases recurred within 2 years after the operation, which is along with the epidemiological data. Among the 121 cases, we discovered 27.27% (33/121) ones with a relatively higher para-cancerous Anillin expression. Notably, 63.6% (21/33) of the cases with a higher Anillin expression at both mRNA and protein stages recurred within 2 years during the follow-up period; And only 7.95% (7/88) cases, suffered from short-term recurrence, showed lower Anillin in para-cancerous tissues, demonstrating almost 75.0% (21/28) recurrence was detected with a higher Anillin in para-cancerous tissues (Fig. 1A, B). RT-qPCR assay indicated that Anillin mRNA remained at a higher level in the recurred cases (Fig. 1C). Along with this, the IHC assay also presented a relatively high expression of Anillin protein in the para-cancerous liver tissues in most of the recurred cases (Fig. 1D).

Meanwhile, we detected the correlation between para-cancerous Anillin expression and the clinicopathological features of those 121 HCC patients. As Table 1 shows, there was no significant correlation observed between Anillin and the age, gender, Tumor encapsulation, Venous invasion, Alpha-fetoprotein (AFP) level, or virus control status. Whereas, a significant positive trend toward larger tumor size, advanced TNM stages, and more incidence of tumor microsatellite formation was discovered in patients with higher para-cancerous Anillin expression. We prompted that the highly expressed Anillin might associate with HCC recurrence connecting with relatively dismal tumor characteristics, such as more active tumor cell proliferation and micro-invasion of the tumor micro-environment.

### High Anillin expression in para-cancerous tissues is related to short-term recurrence according to the GEO database information

**Fig 2:**
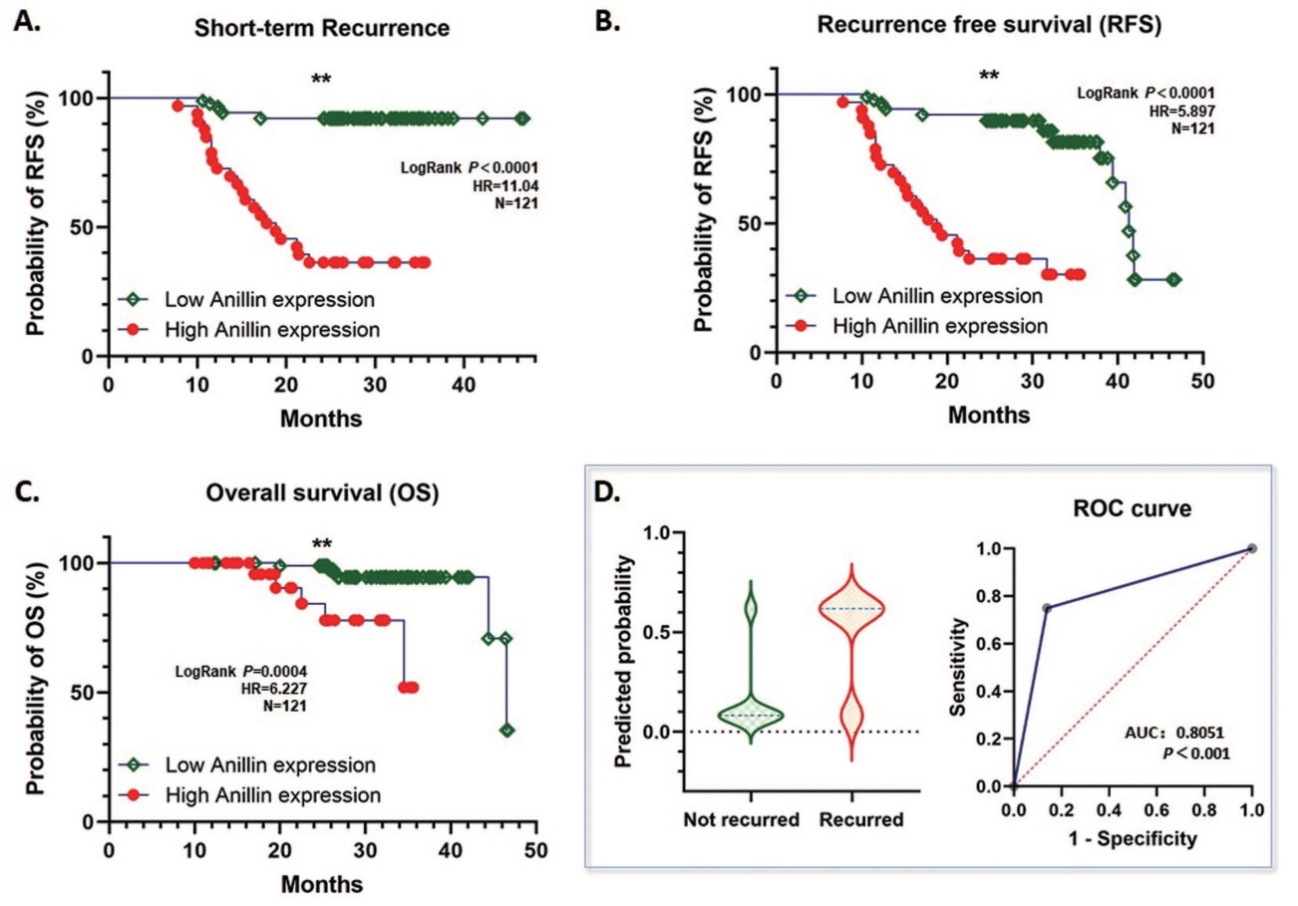
** Correlation between Anillin expression and recurrence-free survival (RFS) and overall survival (OS) of HCC cases.** The follow-up information of the 121 cases of HCC was analyzed. **A.** High Anillin expression in the para-cancerous liver tissues is associated with dismal short-term RFS within 2 years of the HCC patients (**P<0.001). **B.** Highly expressed Anillin is associated with a dismal total RFS during the whole follow-up period (**P<0.001)** C.** High Anillin expression in the para-cancerous liver tissues is significantly associated with a poor the over-all survival (OS) was observed, which further indicated a poorer OS among the patients with a relatively (*P*<0.007).** D.** The logistic regression analysis was conducted. High Anillin expression is significantly correlated with short-term recurrence (OR=18.46; 95%CI: 6.856 to 55.54; AUC: 0.805; *P*<0.001).

According to our follow-up data of the 121 HCC patients, highly expressed para-cancerous Anillin is associated with a dismal short-term recurrence-free survival (RFS) within 2 years (*P*<0.001) (Fig. 2A). Likewise, during the whole follow-up period, even though the total RFS was similar between the high and low Anillin expression group, the short-term recurrence covered most of the relapsed patients with a significant difference, which strongly suggested a possibility of alarming the short-term recurrence after radical surgery (Fig. 2B). And consequentially, a significant difference in the over-all survival (OS) was observed, which further indicated a poorer OS among the patients with a relatively higher Anillin expression in the para-cancerous liver tissues (*P*<0.007) (Fig. 2C). The result of the logistic regression analysis showed that high Anillin expression is significantly correlated with short-term recurrence (OR=18.46; 95%CI: 6.856 to 55.54; AUC: 0.805; *P*<0.001) (Fig. 2D).

**Fig 3:**
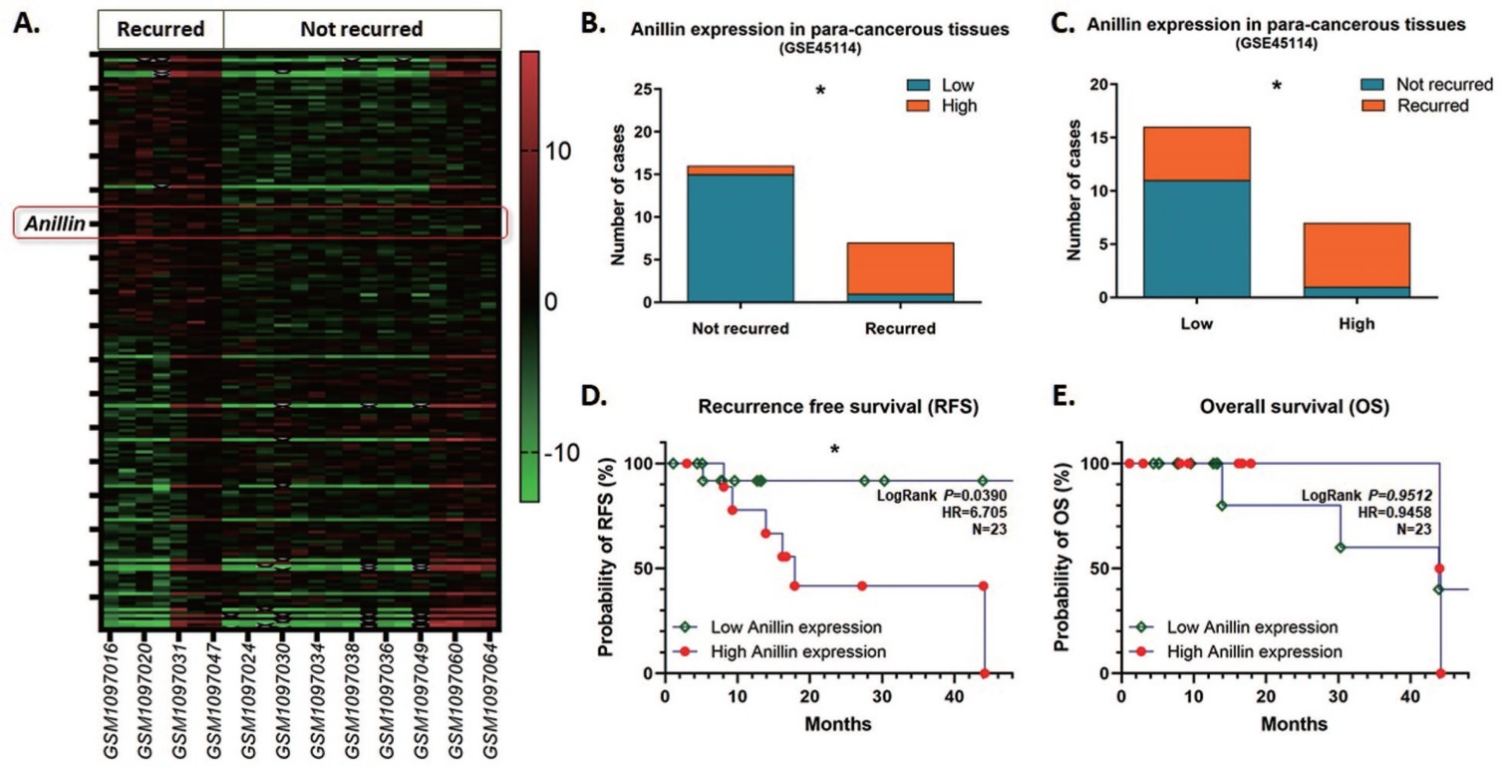
** Anillin expression profile in the para-cancerous liver tissues from the NCBI GEO database.** GSE45114 dataset was selected from the NCBI GEO database for its comprehensive follow-up information of 45 samples of HCC patients along with the para-cancerous liver tissues. **A.** Heatmap was generated according to the top 200 differentially expressed genes (DEGs) with significance in the para-cancerous liver tissues in the GSE45114 dataset. In the recurred patients, Anillin presented to be higher expressed in the para-cancerous liver tissues. **B~C.** Most of the recurrence happened in the patients with a highly Anillin expressed in the para-cancerous tissues (71.43%). **D.** The follow-up data in GSE45114 dataset was analyzed. Higher Anillin expression was associated with lower RFS (*P*<0.05).

As the inclination of recurrence with high Anillin expression in the para-cancerous tissues of the patients in our medical center, we reviewed and explored the GEO database for further validation. Among the tremendous datasets, GSE45114 presents comprehensive follow-up information of 45 samples of HCC specimens and the para-cancerous liver tissues. As observed, 23 HCC clinical cases were described with recurrence information, among which 7 cases recurred and 16 cases did not, and counted a 30.4% recurrence rate. The heatmap presents the top 200 differentially expressed genes (DEGs) with significance in the para-cancerous liver tissues (Fig. 3A), and most of the recurrence happened in the patients with a highly Anillin expressed in the para-cancerous tissues (71.43%; 5/7) (Fig. 3B, C). Simultaneously, combined with the real patient detection of our center, the analysis of the follow-up data in the GSE45114 dataset demonstrated that Anillin is associated with lower RFS (*P*<0.05) (Fig. 3D). Whereas, there seems no significance on OS (Fig. 3E), which was probably because of the relatively small sample size in this dataset.

### Binuclear hepatocytes reduced remarkably in para-cancerous tissue of the patients recurred in short-term

At least 30%∼40% of the hepatocytes in the human liver are polyploid, mainly generated through cytokinesis failure after weaning in the infant stage. Contributed to this mechanism, binuclear hepatocytes compose the major population of the polyploid cells. During tumor initiation, the ratio of polyploid hepatocytes reduces and facilitates tumorigenesis, and the loss of binuclear hepatocytes is a sign of depolyploidization. By detecting the HE staining microscopic images, the binuclear hepatocytes could be counted to present the proportion of polyploidy.

**Fig 4:**
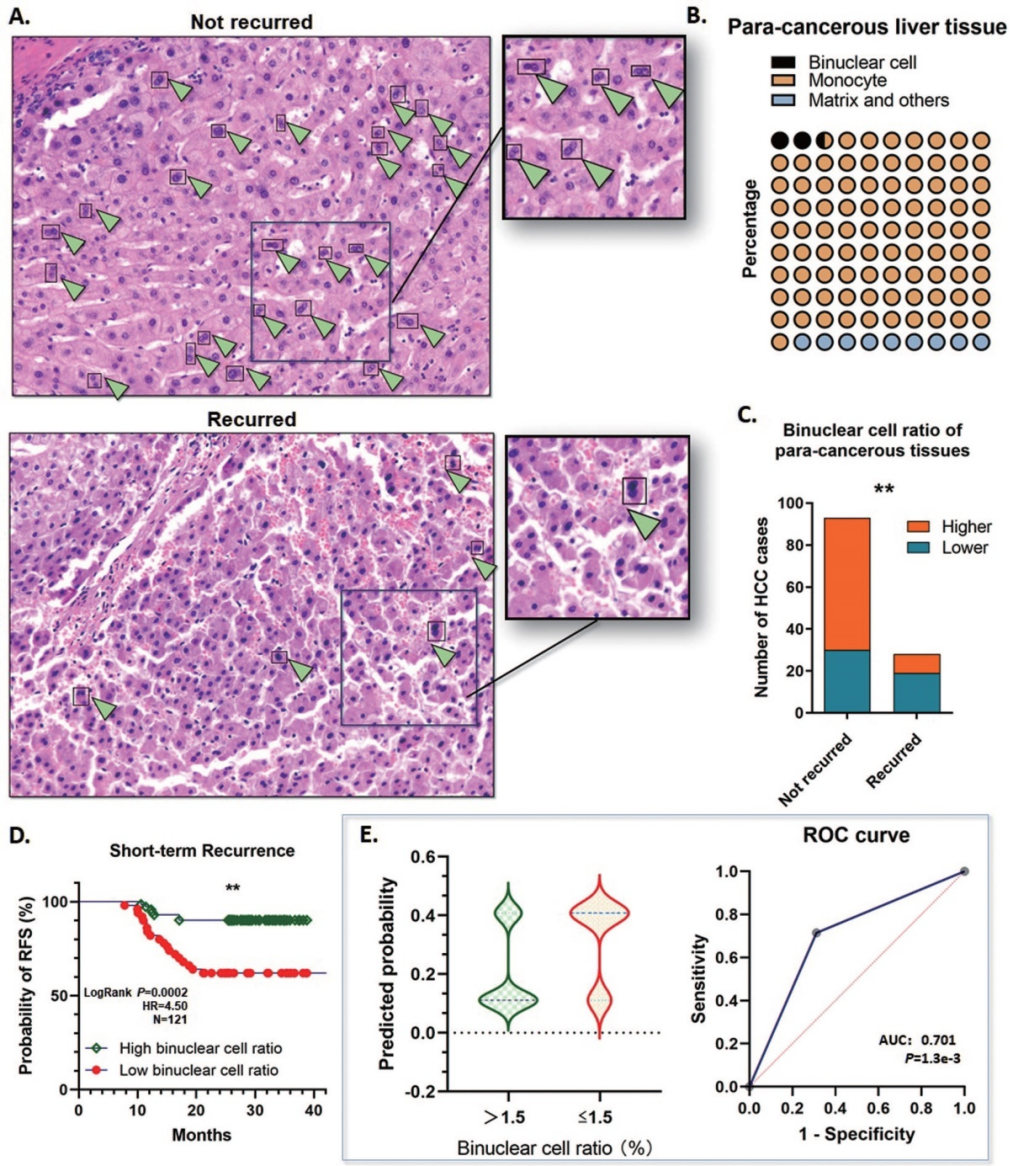
** Binuclear hepatocytes reduced in para-cancerous tissue of the short-term recurred HCC patients. A.** Representative graph of HE staining examination of the para-cancerous liver tissues in the 121 HCC cases. Binuclear hepatocytes reduced significantly in the patients with short-term recurrence. **B.** The proportion of polyploidy was calculated according to the binuclear hepatocytes. The binuclear hepatocytes ratio was ranging from 1.1%~5.25% (Mean: 2.56%; Median: 2.23; SD: 1.322). **C.** 40.50% (49/121) cases contained a ratio of binuclear hepatocytes ≤1.5%. Among the short-term recurred cases, in the para-cancerous liver tissues, 67.9% (19/28) specimens showed a low binuclear hepatocyte ratio <1.5%. On the contrary, in the group not recurred, only 32.95% (29/88) cases showed a relatively lower polyploidy ratio (**P<0.01). **D.** Kaplan-Meier plot was generated. The patients with a lower binuclear cell ratio (≤1.5%) in the para-cancerous tissues showed a significantly lower RFS in the short term (**P<0.01). **E.** The logistic regression analysis was conducted. A lower binuclear cell ratio, ≤1.5%, in the para-cancerous tissues showed a significant correlation with a high risk of short-term recurrence. (OR=5.517; 95%CI: 2.247 to 14.70; AUC: 0.701; *P*=1.3e-3).

On basis of this point, two assigned professional pathologists counted the binuclear hepatocytes in the para-cancerous liver tissues by the range of 1 cm close to the tumor margins. The proportion of polyploidy was calculated according to the binuclear hepatocytes, and the binuclear hepatocytes ratio was ranging from 1.1%~5.25% (Mean: 2.56%; Median: 2.23; SD: 1.322), which indicated that remarkable defection of polyploid hepatocytes is a common event by the early stages of HCC tumorigenesis (Fig. 4A, B). All these cases were divided into the high binuclear cell ratio group and the low one, by applying a cutoff value of 1.5%, and about 40.50% (49/121) cases were determined ≤1.5%. Notably, among the short-term recurred cases, 67.9% (19/28) of para-cancerous specimens showed a low binuclear hepatocyte ratio <1.5%. Whereas in the group not recurred, only 32.95% (29/88) cases showed a relatively lower polyploidy ratio (Fig. 4C). Along with this, a lower binuclear cell ratio in the para-cancerous tissues showed a significant correlation with a high risk of short-term recurrence (OR=5.517; 95%CI: 2.247 to 14.70; AUC: 0.701; *P*=1.3e-3) (Fig. 4D, E). The findings above strongly prompted that depolyploidization in the liver through reducing binuclear hepatocytes is correlated with a high risk of recurrence in the short term.

### Binuclear hepatocytes count is negatively correlated with Anillin expression in para-cancerous liver tissue

**Fig 5:**
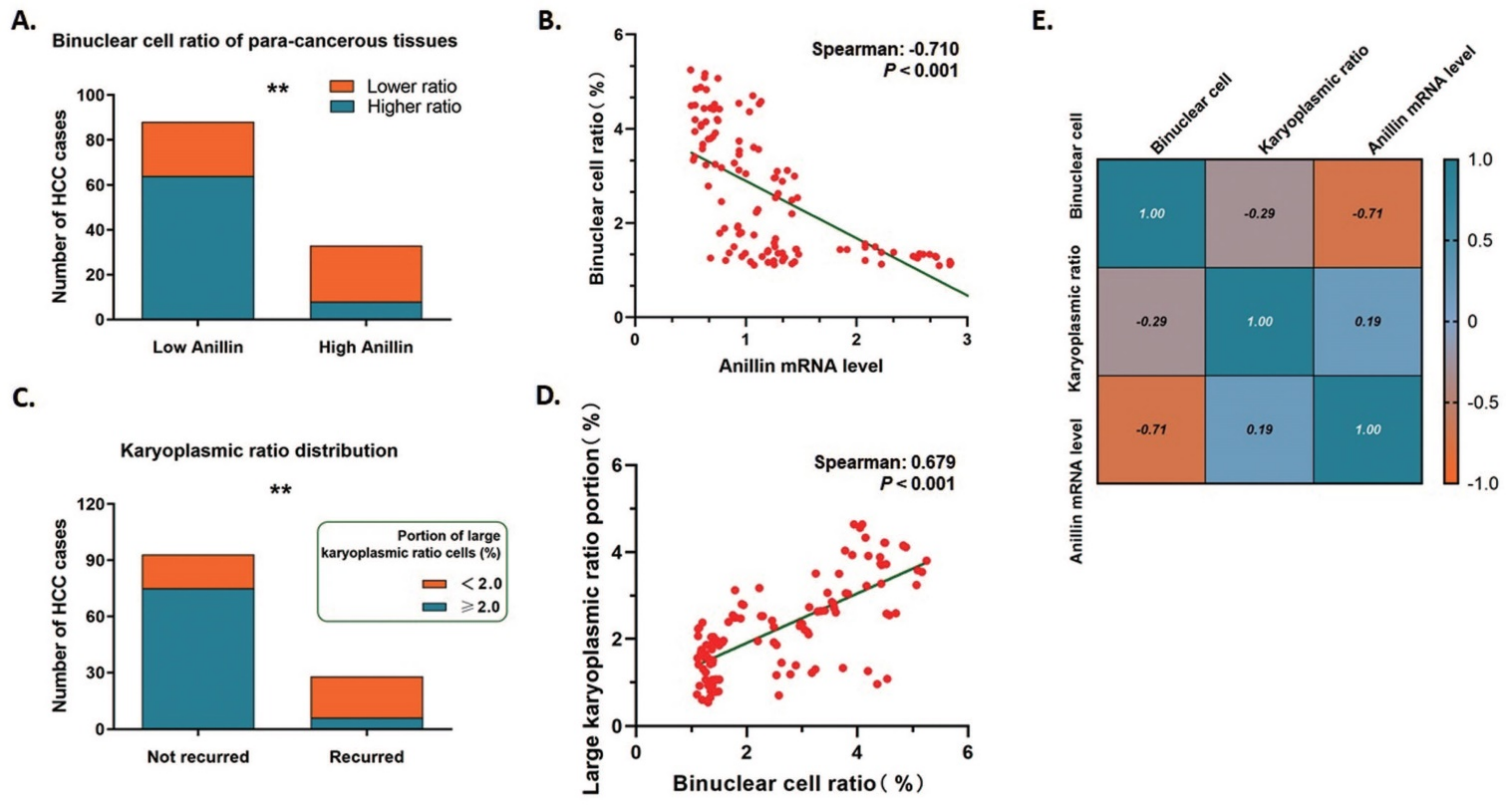
** Binuclear hepatocytes count is negatively correlated with Anillin expression in para-cancerous liver tissues. A.** 75.76% (25/33) of the liver tissues in the group of high Anillin expression presented a remarkable reduction of binuclear hepatocytes by a ratio lower than 1.5%; Only 27.27% (24/88) cases in the group of lower Anillin expression showed a binuclear hepatocyte ration less than 1.5% (**P<0.01). **B.** Spearman correlation analysis was conducted. A negative correlation between Anillin and binuclear hepatocyte ratio was observed (Spearman:-0.710; *P*<0.001). **C.** In short-term recurred cases, only 21.43% (6/28) presented a high portion of large karyoplasmic ratio cell; and in the cases without short-term recurrence, this portion was up to 80.65%(75/93) (**P<0.01). **D.** The large karyoplasmic ratio portion in the para-cancerous tissues showed a significant positive linear correlation with binuclear cell ratio (Spearman: 0.679, *P*<0.001). **E.** Both of binuclear hepatocyte ratio and large karyoplasmic ratio portion were negatively correlated with higher expression of Anillin in the para-cancerous liver tissue.

Anillin is the critical regulating gene controlling cytokinesis, and we wondered if the expression status of Anillin in the para-cancerous tissues exactly affected the binuclear hepatocytes ratio. 75.76% (25/33) of the liver tissues in the group of high Anillin expression presented a remarkable reduction of binuclear hepatocytes by a ratio lower than 1.5%; On the contrary, the cases with this ratio of less than 1.5% in the group of lower Anillin expression is only 27.27% (24/88) (Fig. 5A). Spearman correlation analysis demonstrated a negative correlation between Anillin and binuclear hepatocyte ratio, and further indicated the value of recognition of Anillin-related binuclear cells in predicting early HCC recurrence (Spearman:-0.710, *P*<0.001) (Fig. 5B).

The karyoplasmic ratio is a proportion of the nucleus to the cytoplasm of a cell in volume. For any single cell, the karyoplasmic ratio is altered during cell cycles along with the changes in the nucleus and cytoplasm volume, and is normally around 1:1.6 (0.625). Polyploid hepatocytes are generated through cytokinesis failure, which specifically forms a polyploid cell containing a 2-fold volume of nucleus and makes the karyoplasmic ratio increase. We measured the karyoplasmic ratio within the range of 1 cm close to the tumor margins, and counted the number of cells with a large karyoplasmic ratio (0.8 as a cutoff value). Interestingly, only 21.43% (6/28) shortly recurred cases were observed containing a high portion of large karyoplasmic ratio cells; While this portion made up 80.65% (75/93) of the cases without short-term recurrence on the contrary (Fig. 5C). And as expected, the large karyoplasmic ratio portion in the para-cancerous tissues showed a significant positive linear correlation with the binuclear cell ratio (Spearman: 0.679, *P*<0.001) (Fig. 5D). Simultaneously, both of these two pathologically observed parameters were correlated with higher expression of Anillin negatively as Fig. 5E showed. The findings above also strongly demonstrated the effect of Anillin on cell ploidy status and the relative short-term recurrence.

## Discussion

Approximately, the recurrence of HCC after surgery is up to 60%~70% and mostly occurred in the short-term 17. Patients who recurred in the short-term usually possessed aggressive tumor pathological characteristics, like large tumor size, multiple tumors, poor cell differentiation, and microscopic vascular invasion 18-20. Numerous evidence indicated that even conducting the complete R0 resection or ablation, the disease would recur and progress into incurable stages because of the occult carcinogenic microenvironment in the remnant liver. However, it is difficult for researchers to identify reliable and credible risk factors for short-term recurrence of HCC, which is of vital importance for figuring out individual adjuvant therapies in preventing tumor progress and achieving ideal clinical outcomes after surgery.

As a fascinating feature of liver parenchyma, hepatic polyploidy presents various compositions (diploid, tetraploid, octoploid nucleus) depending on different species and mechanisms of polyploidization 8, 21. In rodents, the main hepatocytes are tetraploid (binuclear with two diploid nuclei or mononuclear with one tetraploid nucleus) and octoploid, and compose up to 90% of the hepatocytes 22. Whereas, in the human liver, hepatocytes gain polyploidy characteristics during postnatal development through a scheduled cytokinesis failure, which mainly generates binuclear tetraploid hepatocytes with two diploid nuclei, and shows no specific intrahepatic parenchyma zonation 23. According to the previous works of literature, there are several methods to recognize the polyploid cells in the laboratory, including karyotype identification, whole-genome sequencing, and fluorescence in situ hybridization. However, we are seeking an innovative method, that could be utilized in the clinic easily, effectively, and with high-cost performance.

We propose that the characteristic of human hepatic polyploidy provides us a possibility to recognize the polyploid hepatocytes by counting the specific binuclear parenchymal cells in the liver. On basis of this, we focused our attention on the HE staining observation, which is the most common method for pathological examination clinically. We assigned two experienced pathologists to detect the HE stained specimens of the para-cancerous liver tissue for a precise counting of the binuclear hepatocyte, with the exclusion of the interstitial cells and connective tissue. On rely of the strict quality control, we effectively distinguished the above cell types and calculate the proportion of binucleate cells in hepatocytes. According to the convincible literature, the hepatic polyploidy status changes dynamically under stresses like excessive metabolic workload, DNA damage, and chemical-induced liver injury 24-26. This means that the hepatic ploidy profile could be a useful index for accessing the pathophysiological status of the liver.

HCC is derived from hepatocytes through characteristic somatic DNA mutation and chromosomal abbreviation. Notably, even if polyploid generation is associated with tumorigenesis in most human malignancies, it is quite opposite for the liver between HCC carcinogenesis and hepatic polyploidy. Zhu's laboratory 11 has described a pivotal discovery that, in various carcinogen-driven models, polyploid hepatocytes acquire much more loss-of-function events to bypass the protection mechanism against HCC transformation than the diploid liver cells. And also, the binuclear polyploid hepatocytes are observed barely exist in human HCC tissues, which suggests that reduction of cytokinesis failure and hepatocyte depolyploidization should happen throughout the whole process of HCC initiation and progress 7, 27. And in this ploidy-related process, the E2F7/8 signaling pathway has been reported as an effective HCC suppression mechanism via inhibiting cell cycle-dependent gene transcription and maintaining hepatic polyploidy by impacting cellular proliferation and development 28. Mice deficient in E2F7/8 were found to lose the characteristic of hepatic polyploidy with numerous diploid hepatocytes in the liver, and remarkably develop spontaneous HCC 29. Given this finding, the correlation between binuclear polyploid hepatocytes and patients' short-term recurrence comes to be a probable breach for evaluating HCC outcomes.

Notably, during the process of the cell cycle, Anillin was also found as an evolutionarily conserved regulator exerting critical function on promoting cytokinesis to generate two daughter cells at the end stage of mitosis, and knock-out Anillin sequentially blocked hepatocyte division, generated binuclear hepatocytes, and effectively reduced depolyploidization derived liver tumor development 15, 30, 31. In knowledge, Anillin is remarkably over-expressed in various solid malignancies, including breast, pancreatic, lung, and gastric, and is commonly associated with poor outcomes in the patients 32-34. For HCC, consistent with these findings, our previous study has already verified that Anillin was over-expressed in HCC and facilitated both tumor cell proliferation and tumor growth 35. Similarly, at about the same time, our findings are also matched with some of the other authors' discoveries on the general over-expression of Anillin in HCC and its promoting effects on either tumorigenesis or development 36, 37.

Based on the evidence above, we believe that Anillin plays an important role as an oncogene in HCC. However, all these studies are focusing on the Anillin right in the tissues of the tumor, and the detection of the para-cancerous tissues is quite limited. In accordance, with this study, besides the discovery in HCC specimens, we furtherly detected the Anillin expression profile in the para-cancerous liver tissue. Although the para-cancerous tissues generally showed a lower Anillin expression level than the tumor specimens, there exists a significant difference between the patients who recurred in short term or not. Most of the recurrence occurred in patients with relatively high para-cancerous Anillin expression. Meanwhile, the ratio of binuclear hepatocytes in the para-cancerous tissue showed a similar correlation with Anillin. All the findings suggested Anillin as a potent symbol for reflecting the status of hepatic ploidy, which is correlated with short-term recurrence. On basis of the above, we analyzed and confirmed the significant positive relationship between high Anillin expression, a decrease of binuclear hepatocytes in the para-cancerous liver, and also the incidence of short-term HCC recurrence.

To further support the correlation between binuclear hepatocyte ratio with hepatic ploidy status and HCC recurrence, we additionally calculated the karyoplasmic ratio of the hepatocytes of the para-cancerous tissues as another reference approach. As our observation shows, binuclear hepatocytes presented a significantly higher karyoplasmic ratio, which is up to a value larger than 0.8. And the proportion of cells with the karyoplasmic ratio of this cutoff value also validated a positive correlation with either Anillin expression level or the recurrence of HCC. This strongly supports our point that Anillin facilitates hepatic depolyploidization and is associated with short-term recurrence of HCC.

**Fig 6:**
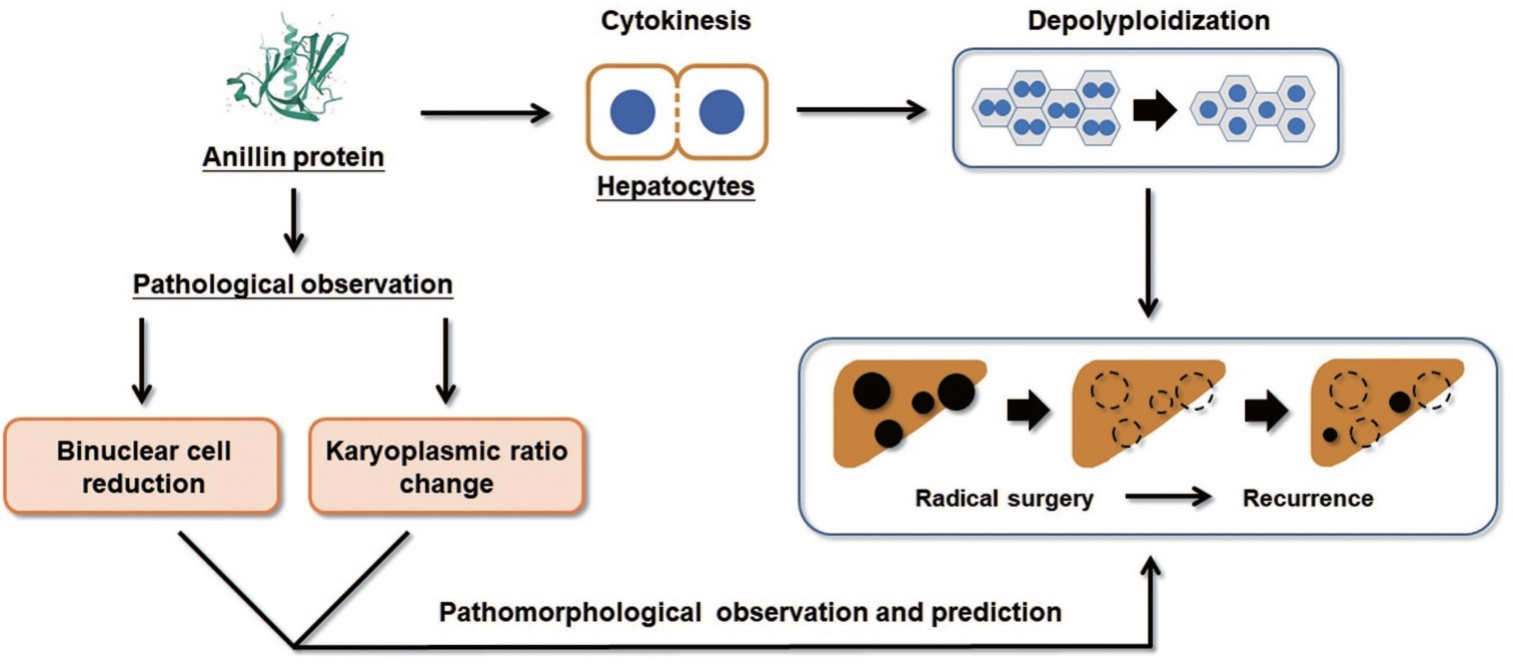
** Binuclear hepatocytes count is negatively correlated with Anillin expression in para-cancerous liver tissues.** The summary of the correlation between Anillin expression, binuclear hepatocyte ratio, karyoplasmic ratio distribution in the para-cancerous liver tissues, and HCC short-term recurrence after radical surgery.

Collectively, our study proposes that the raising of Anillin in hepatocytes is a continuous event that occurs in the early stage of liver carcinogenesis and maintains throughout the progress of HCC (Fig. 6). The change of Anillin is significantly correlated with the depolyploidization of hepatocytes, by which the binuclear polyploid hepatocytes extremely decreased, and the portion of large karyoplasmic hepatocytes defected. The para-cancerous Anillin measurement and these two related pathomorphological examinations above are a mighty strategy for screening high risk of HCC recurrence, and would be valuable for designing individual HCC adjuvant treatment post-operation.

## Supplementary Material

Supplementary table.

## Figures and Tables

**Table 1:** ** Correlation between Anillin expression in the para-cancerous liver tissues of 121 HCC cases and clinicopathologic features.** Anillin transcript level associated with clinicopathologic features in the para-cancerous liver tissues of 121 HCC patients cases, including age, gender, tumor size, tumor stage (AJCC), tumor encapsulation, tumor microsatellite formation, vein invasion, HBsAg status, AFP level, and liver cirrhosis. Statistically, significance was assessed by Fish's exact text.

Clinicopathologic parameters	Anillin expression in para-cancerous tissues		P*
	Lower (n=88)	Higher (n=33)	
Age (years)			
≤50	48	17	0.839
>50	40	16	
Gender			
Male	52	18	0.683
Female	36	15	
Diameter (cm)			
≤5	36	26	0.0002
>5	52	7	
TNM stage			
I~II	32	23	0.0018
III~IV	56	10	
Tumor encapsulation			
Absent	47	25	0.220
Present	41	8	
Tumor microsatellite formation			
Absent	49	26	0.022
Present	39	7	
Venous invasion			
No	61	26	0.368
Yes	27	7	
HBsAg			
Negative	8	1	0.442
Positive	80	32	
AFP(ng/ml)			
≤400	21	12	0.117
>400	67	21	
Cirrhosis			
Absent	8	5	0.339
Present	80	28	
