# Supplementary material for: Raising of Anillin expression in para-cancerous hepatocytes is associated with hepatic depolyploidization and short-term recurrence of hepatocellular carcinoma after radical operation
Source: J Cancer. 2022 May 29;13(9):2729–39. doi: 10.7150/jca.72890 (PMC9254876; doi:10.7150/jca.72890)
Supplement: Supplementary file 1 — Supplementary table. [file jcav13p2729s1.pdf]

Supplementary materials

Suppl. Table. 1

The primers for RT-qPCR assay

Genes	Forward	Reverse
**Anillin Primers-01**	**5’-ACAAGAACAGCCTGGTACCG-3’**	**5’-TTCAGGTCGAGCATCGTGTG-3’**
**Anillin Primers-02**	**5’-GTGTGCTACGAGCTGGACTT-3’**	**5’-GCCATGGTCCGACAAGAACA-3’**
